# High cholesterol absorption efficiency enhances proatherogenic properties of low‐density lipoprotein particles

**DOI:** 10.1111/joim.70085

**Published:** 2026-04-02

**Authors:** Katariina Öörni, Lauri Äikäs, Maija Ruuth, Feven Tigistu‐Sahle, Reijo Käkelä, Ingmar Wester, Helena Gylling, Piia Simonen

**Affiliations:** ^1^ Atherosclerosis Research Laboratory Wihuri Research Institute Helsinki Finland; ^2^ Molecular and Integrative Biosciences Faculty of Biological and Environmental Sciences University of Helsinki Helsinki Finland; ^3^ Helsinki University Lipidomics Unit, HiLIPID Helsinki Institute of Life Science, HiLIFE, and Biocenter Finland Helsinki Finland; ^4^ Raisio Group plc Raisio Finland; ^5^ Heart and Lung Center, Cardiology Helsinki University Hospital and University of Helsinki Helsinki Finland

**Keywords:** atherosclerosis, atherosclerotic cardiovascular disease, cholesterol absorption, cholesterol metabolism, lipidomics, low‐density lipoprotein aggregation, proteoglycan‐binding

## Abstract

**Background and Aims:**

High cholesterol absorption efficiency is determined by genetic variation in small intestinal sterol transporters and affects one‐third of individuals. Their risk for atherosclerotic cardiovascular disease (ASCVD) is increased compared with low cholesterol absorbers, despite similar serum lipid concentrations. We investigated the association of cholesterol absorption efficiency with proatherogenic properties of low‐density lipoproteins (LDLs).

**Methods:**

The study cohort consisted of 90 middle‐aged participants, 56 females and 34 males, without lipid‐lowering therapy or ASCVD. They were divided into low (*n* = 45) and high (*n* = 45) cholesterol absorbers by the median value of serum cholestanol to cholesterol ratio. LDL aggregation susceptibility and binding of lipoproteins to proteoglycans were determined as biomarkers of lipoprotein atherogenicity. Nuclear magnetic resonance spectroscopy, mass spectrometry, and gas–liquid chromatography were used to determine lipoprotein subclass profile, LDL lipidome, and serum concentrations of cholesterol and noncholesterol sterols, respectively.

**Results:**

Age, dietary cholesterol, and serum total cholesterol or lipoprotein cholesterol levels did not differ between the groups. In the high absorbers, LDL particles were larger, and LDL aggregation susceptibility and the binding of lipoproteins to proteoglycans were increased in the low absorbers. Of LDL surface lipids, sphingomyelin 42:3;O2, and phosphatidylcholine (PC) 32:0 correlated positively, whereas PC 32:1 correlated negatively with serum cholestanol levels. These LDL surface lipids were also associated with increased LDL aggregation susceptibility and proteoglycan‐binding.

**Conclusions:**

The present findings suggest that increased proatherogenic properties in high cholesterol absorbers may contribute to increased ASCVD risk.

**Registration:**

https://clinicaltrials.gov/study/NCT01315964.

## Graphical abstract

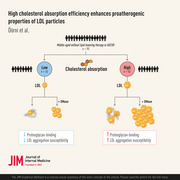

**Abbreviations**
ABC: adenosine triphosphate‐binding cassette
apo: apolipoprotein
ASCVD: atherosclerotic cardiovascular disease
CE: cholesteryl ester
CETP: cholesteryl ester transfer protein
GLC: gas–liquid chromatography
HDL‐C: high‐density lipoprotein cholesterol
LDL: low‐density lipoprotein
LDL‐C: LDL‐cholesterol
LPC: lysophosphatidylcholine
MS: mass spectrometry
NMR: nuclear magnetic resonance
PC O‐: ether‐linked phosphatidylcholine
PC: phosphatidylcholine
PIS: precursor ions scans
SM: sphingomyelin

## Introduction

Atherosclerotic cardiovascular diseases (ASCVDs) are the most common causes of deaths worldwide. The root cause of ASCVDs is retention and accumulation of low‐density lipoprotein (LDL)–derived cholesterol and other lipids in the arterial wall. Decreasing the number of circulating LDL particles and LDL‐cholesterol (LDL‐C) concentration in circulation decreases the risk of ASCVD events [1, 2, 3]. There are, however, also other factors that can contribute to the development of atherosclerosis. In the following, we focus on three variables with potential atherogenicity, that is, high cholesterol absorption efficiency, the retention of lipoproteins by arterial wall proteoglycans, and the modification and aggregation of LDL particles.

The atherogenicity of circulating LDL particles and other apolipoprotein (apo) B–containing lipoproteins depends on their ability to be retained by intimal proteoglycans and by their susceptibility to become modified and aggregated in the arterial wall [4, 5]. Both qualities of lipoproteins are influenced by their lipid composition and have been linked to the risk of future ASCVD events [6, 7, 8, 9]. Importantly, these properties of lipoproteins are modifiable independently of changes in LDL‐C and are increased, for example, by saturated fat intake, whereas unsaturated fat intake, *n*‐3 polyunsaturated fatty acid supplementation, and phytostanol ester consumption as part of heart‐healthy diets change lipoprotein lipid composition and reduce their binding to proteoglycans or their aggregation susceptibility [6, 8, 10, 11]. In mouse models of atherosclerosis, the inhibition of either proteoglycan‐binding of plasma lipoproteins or LDL aggregation reduces atherosclerosis [6, 12, 13], findings suggesting a causal role of these processes in atherogenesis.

Cholesterol absorption efficiency is considered high when exceeding 50%–60% [14, 15]. Approximately 30% of individuals are high cholesterol absorbers according to evaluation with absolute dual‐isotope feeding methods [14, 15]. Genetic variations causing loss of function of the intestinal sterol transporters adenosine triphosphate‐binding cassette proteins ABCG5 and ABCG8 increase cholesterol absorption efficiency due to the deficient returning of cholesterol to intestinal lumen [16, 17, 18, 19]. The risk of developing atherosclerotic lesions and ASCVD is increased in high cholesterol absorbers when compared to those having low cholesterol absorption efficiency [20, 21, 22, 23, 24]. Importantly, increased ASCVD risk is not explained by differences in circulating cholesterol concentrations between high and low cholesterol absorbers, and the underlying mechanism(s) in the increased ASCVD risk of the high cholesterol absorbers is unknown.

Here, in this post hoc study of our original randomized, controlled, double‐blind, and parallel clinical intervention [25, 26], the aim was to evaluate the potential association of high cholesterol absorption with LDL particle lipidome, proteoglycan‐binding, or LDL aggregation susceptibility. The study population was divided into two groups based on the median value of baseline serum cholestanol to cholesterol ratio, a validated serum biomarker of cholesterol absorption efficiency [27]. We now determined LDL lipidomes, LDL aggregation susceptibility, and proteoglycan‐binding from these two groups called low‐ and high‐cholesterol absorbers.

## Patients and methods

### Study population

The original intervention included 92 participants who completed the 6‐month study called BLOOD FLOW in 2011 [25]. The participants were mildly to moderately hypercholesterolemic office employees in good and stable overall health and without ASCVD. No inclusion criteria were set for serum and lipoprotein lipids, but lipid‐lowering medication or the consumption of nutrient supplements interfering with serum cholesterol concentration, such as red rice or berberine, were exclusion criteria. In case of consumption of phytosterol/phytostanol products, the participants could be included in the study after a 3‐week washout period. In 2018, the participants were recontacted for permission to use their frozen sera for exploratory analyses in order to evaluate the aggregability of LDL and the binding of serum lipoprotein‐derived cholesterol to human aortic proteoglycans during phytostanol ester consumption [8].

The present study population, *n* = 90, comprises the study population from the earlier studies [8, 25, 26] apart from two persons (of 92), who were excluded because of missing data. The age range was 24–66 years with a mean of 50.9 ± 1.0 years (SEM). Thirty‐four participants were men and 56 were women.

All participants provided written informed consent. The study was conducted in accordance with the Declaration of Helsinki. The original study protocols were approved by the HUS Regional Committee on Medical Research Ethics and Uusimaa Hospital District (Helsinki, Finland) in 2011 and 2018. The original clinical study was registered in clinicaltrials.gov as NCT01315964.

### Study design

In the original 6‐month trial, the participants started the randomized periods using rapeseed oil margarine without (control group) or with added phytostanol esters (phytostanol group: 3.0 g phytostanols/day) [25]. Blood samples were drawn after a 12‐h fast at baseline and at the end of the study. In the present study, only the baseline data are included [8, 27, 28, 29, 30].

### Methods

Anthropometric measurements and inclusion in the study were determined by a physician. Dietary information was collected by a nutritionist. Circulating lipid profiles, including serum total cholesterol, LDL‐C, high‐density lipoprotein cholesterol (HDL‐C), and serum triacylglycerol (TGs), were analyzed using standardized automated analyzer systems at the Central Laboratory of Helsinki University Hospital (HUSLAB). Non‐HDL‐C was calculated.

### Gas chromatography analyses of circulating sterols

Serum concentrations of total cholesterol, the noncholesterol sterols desmosterol and lathosterol (precursors of cholesterol), cholestanol (a metabolite of cholesterol), and campesterol and sitosterol (phytosterols) were quantified with capillary gas–liquid chromatography (GLC) (Agilent 7890GC System, Agilent Technologies) equipped with a 50‐m long Ultra 2 capillary column (5% phenyl‐methyl siloxane) (Agilent Technologies) and flame ionization detection with 5α‐cholestane as the internal standard [31]. Serum concentrations of the noncholesterol sterols were adjusted to that of cholesterol in the same GLC run and expressed as ratios to cholesterol (×10^2^ µmol/mmol of cholesterol) and called ratios herein. As ratios to cholesterol, desmosterol and lathosterol are validated biomarkers of relative whole‐body cholesterol synthesis, whereas campesterol, sitosterol, and cholestanol are validated biomarkers of cholesterol absorption efficiency, all assayed with the absolute methods or genetic variants of cholesterol metabolism [27, 28, 29, 30]. The prerequisites for assaying cholesterol metabolism, whether by relative or absolute methods, include maintaining stable dietary and lifestyle habits to ensure steady‐state cholesterol metabolism.

### NMR‐derived lipoprotein subclass and size

Plasma lipoprotein subclass concentrations and particle sizes were determined by proton nuclear magnetic resonance (NMR) spectroscopy using the LipoScience (LabCorp) NMR LipoProfile platform. The analysis quantifies concentrations of very low‐density lipoprotein (VLDL), LDL, and high‐density lipoproteins (HDLs) particles, along with their major subclasses: large (L), medium (M), small (S), and very small (XS) particles (e.g., L‐VLDL, M‐LDL, S‐HDL). Mean particle diameters for VLDL, LDL, and HDL are also obtained from the data.

### Measurement of lipoprotein binding to proteoglycans

Proteoglycans were isolated from human aortas [32] and used to coat 96‐well plates overnight at 4°C. The plates were blocked for 1 h at 37°C with PBS containing 3% BSA, 1% fat‐free milk, and 0.05% Tween 20. Serum samples (1 µL) were diluted in 100 µL of 20 mmol/L MES, 140 mmol/L NaCl, 2 mmol/L CaCl_2_, and 2 mmol/L MgCl_2_ pH 5.5 and incubated in the wells for 1 h at 37°C. Unbound lipoproteins were removed by washing with the same buffer containing 50 mmol/L NaCl, after which the amount of bound lipoproteins was determined by measuring the cholesterol in the wells with the Amplex red cholesterol kit (Thermo Scientific).

### Measurement of LDL aggregation susceptibility

LDL particles (*d* = 1.019–1.063 g/mL) were isolated from frozen serum samples using sequential ultracentrifugation [11]. The concentrations of LDL were determined as their protein concentration measured with the Pierce BCA Protein Assay Kit (Thermo Scientific). LDL was diluted to 200 µg/mL in 20 mM MES, pH 5.5 containing 150 mmol/L NaCl and 50 µmol/L ZnCl_2_ and incubated with in‐house–produced human recombinant sphingomyelinase in a microplate [33]. Under these conditions, LDL forms very large aggregates, which were determined using Wyatt DynaPro Plate Reader II (Wyatt Technology). Aggregate size data were collected Dynamics V7 software (Wyatt Technology. Time versus aggregate size curves were used to determine two variables of aggregation susceptibility: aggregate size after incubation for 2 h and the inflection point of the curves. Large aggregate size at 2 h and low inflection point indicate fast aggregation.

### Mass spectrometry analyses of LDL lipidome

LDL lipids were extracted using the Folch method [34]. Lipid extracts were dissolved in chloroform/methanol (1:2 v/v) and spiked with a quantitative internal standard mix (SPLASH LIPIDOMIX Mass Spec Standard No. 330707; Avanti Polar Lipids, Inc.). Before mass spectrometry (MS), NH_4_OH (to give 1% solution) was added to support ionization and prevent sodium adduct formation. Samples were introduced into the electrospray ionization source of a triple quadrupole MS (Agilent 6410 Triple Quad LC/MS; Agilent Technologies, Inc.). MS+ and MS/MS precursor ion scans (PIS) were used to detect TG ([M + NH_4_]^+^) and phospholipid (PIS *m/z* 184), and cholesteryl ester (CE) species (PIS *m/z* 369), respectively. Data were analyzed using MassHunter software (Agilent Technologies, Inc.), and lipid species were quantified and converted to molar percent data using the internal standards and Lipid Mass Spectrum Analysis software [35] and expressed as percentages of surface and core lipids.

### Statistics

Statistical analyses were performed by using IBM SPSS for Windows 22.0 software (IBM SPSS) and GraphPad Prism 10. Sample size estimation (*α* = 0.05 and *β* = 0.20) indicated that the study population was adequate. Normality and homogeneity of variance assumptions were checked before further analyses. Continuous and normally distributed and homogenous variables were tested by using Student's *t*‐test and univariate analysis of variance with Dunnett's T3 multiple comparisons test. Noncontinuous variables were analyzed by using the chi‐square test or Fisher's exact chi‐square test. Variables not normally distributed or non‐homogenous were either transformed logarithmically or tested by using Kruskal–Wallis test with Dunn's multiple comparisons test. Spearman's rank correlation coefficients were calculated for some variables of interest. A two‐sided *p*‐value of <0.05 was considered statistically significant.

## Results

### Serum noncholesterol sterol ratios

**Fig. 1:**
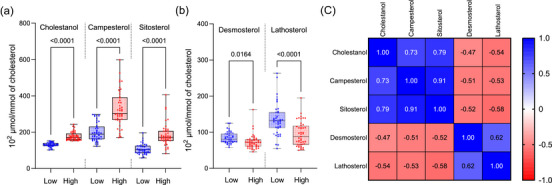
**Biomarkers of cholesterol absorption and synthesis**. The participants (n = 90) were divided into two groups having low and high cholesterol absorption efficiency based on their median value of serum cholestanol to cholesterol ratio. Low cholesterol absorption efficiency: serum cholestanol to cholesterol ratio <151.8 × 10^2^ µmol/mmol of cholesterol; high cholesterol absorption efficiency: ≥151.8 × 10^2^ µmol/mmol of cholesterol. (a) Cholesterol absorption markers. (b) Cholesterol synthesis markers. (c) Spearman's correlation of the cholesterol absorption and synthesis markers (p < 0.0001 in each case).

The participants were divided into two groups based on their cholestanol to cholesterol ratios. Cholestanol is the most stable cholesterol absorption marker, as it is mostly of endogenous origin and only small fluctuations are induced by diet. Here, the individuals with low cholesterol absorption efficiency had serum cholestanol to cholesterol ratio <151.8 × 10^2^ µmol/mmol of cholesterol, and individuals with high cholesterol absorption efficiency had ≥151.8 × 10^2^ µmol/mmol of cholesterol, respectively. As expected, the campesterol and sitosterol to cholesterol ratios, two other markers of cholesterol absorption efficiency, were also higher in the high versus low cholesterol absorbers (Fig. 1a). In contrast, serum biomarker ratios of cholesterol synthesis, the ratios of desmosterol and lathosterol to cholesterol, were significantly lower in the high versus the low cholesterol absorbers (Fig. 1b). The synthesis and absorption biomarkers inversely correlated with each other, indicating that cholesterol metabolism was in steady‐state and the biomarkers were valid (Fig. 1c).

### Demographics, anthropometrics, and dietary cholesterol and fat intakes

The number of males and females, 17 and 28, was the same in the low and high cholesterol absorbers (Table 1). The mean age did not differ between the groups or between the sexes, but body mass index and waist circumference were significantly higher in the low vs. high cholesterol absorbers. Thus, in the low cholesterol absorbers, the body mass index varied from 20.3 to 36.6 kg/m^2^, and six persons were obese (body mass index ≥ 30 kg/m^2^), whereas in the high cholesterol absorbers, the respective figures were from 17.9 to 33.2 kg/m^2^, and two persons were obese. The diets of the participants were tracked with the help of a registered dietitian. The groups had similar nutritional intakes, and no differences were observed in dietary cholesterol. The overall dietary fat was similar between the groups; however, saturated fat intake was significantly higher in the low versus high cholesterol absorbers (Table 1). Data on the menopausal status, the use of oral contraceptives, or hormone replacement therapy were not available. However, the ages of women or men did not differ between the high and low cholesterol absorbers.

**Table 1:** Demographics, anthropometrics, dietary cholesterol and fat intake, serum and lipoprotein lipids, and serum noncholesterol sterol ratios to cholesterol in individuals divided into low and high cholesterol absorption efficiency.

Variables	Low cholesterol absorption (*n* = 45)	High cholesterol absorption (*n* = 45)	*p*‐value
Males/Females, *n*	17/28	17/28	NS
Age, years	52.2 ± 1.29	49.5 ± 1.53	0.095
Body mass index, kg/m^2^	26.3 ± 0.52	23.9 ± 0.50	<0.001
Waist circumference, cm	91.5 ± 1.53	85.8 ± 1.49	0.005
**Dietary cholesterol and fat intake**			
Cholesterol, mg/day	232 ± 14.8	210 ± 13.6	0.142
Fat, g/day	73.1 ± 3.07	71.9 ± 3.63	0.401
Fat, % of energy	34.7 ± 0.98	33.4 ± 0.97	0.187
SFA, % of energy	12.1 ± 0.44	10.7 ± 0.47	0.018
MUFA, % of energy	11.8 ± 0.50	11.8 ± 0.48	0.485
PUFA, % of energy	5.40 ± 0.26	5.88 ± 0.29	0.109
**Serum and lipoprotein lipids**			
Serum cholesterol, mmol/L	5.48 ± 0.13	5.58 ± 0.14	0.296
LDL‐cholesterol, mmol/L	3.47 ± 0.14	3.59 ± 0.11	0.253
Non‐HDL‐cholesterol, mmol/L	3.73 ± 0.15	3.77 ± 0.13	0.425
HDL‐cholesterol, mmol/L	1.75 ± 0.08	1.81 ± 0.06	0.256
Serum triglycerides, mmol/L	1.04 ± 0.07	0.81 ± 0.05	0.007
**Serum noncholesterol sterol ratios to cholesterol** ^a^			
**Biomarkers of cholesterol synthesis**			
Serum desmosterol	84.5 ± 2.32	73.2 ± 2.92	0.016
Serum lathosterol	135 ± 6.37	94.7 ± 4.93	<0.001
**Biomarkers of cholesterol absorption**			
Serum campesterol	200 ± 7.47	327 ± 14.3	<0.001
Serum sitosterol	106 ± 4.08	184 ± 9.00	<0.001
Serum cholestanol	131 ± 1.75	178 ± 3.37	<0.001

*Note*: Mean ± SEM. Low and high cholesterol absorption efficiency: divided by the median value of serum cholestanol to cholesterol ratio at baseline. Low cholesterol absorption efficiency: serum cholestanol to cholesterol ratio <151.8 × 10^2^ µmol/mmol of cholesterol, high cholesterol absorption efficiency: ≥151.8 × 10^2^ µmol/mmol of cholesterol.

Abbreviations: HDL, high‐density lipoprotein; LDL, low‐density lipoprotein; MUFAs, monounsaturated fatty acids; non‐HDL‐cholesterol, serum cholesterol minus HDL‐cholesterol concentration; PUFA, polyunsaturated fatty acids; SFA, saturated fatty acids.

^a^ = ×10^2^ µmol/mmol of cholesterol.

### Serum and lipoproteins lipid profiles

**Fig. 2:**
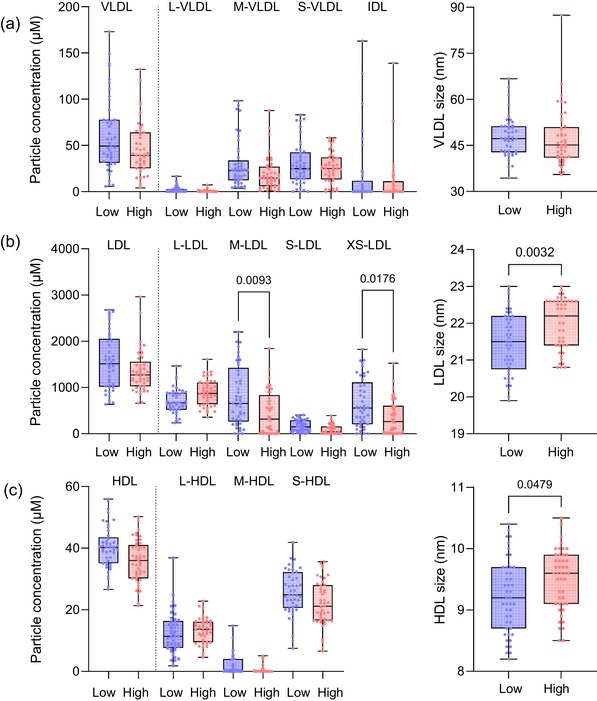
**Lipoprotein particle concentrations and sizes**. Concentrations of lipoproteins and their subclasses and sizes were determined by NMR spectroscopy and are shown separately for low and high cholesterol absorbers: (a) very low‐density lipoprotein (VLDL), (b) low‐density lipoprotein (LDL), and (c) high‐density lipoprotein (HDL). Particle concentration data were analyzed using Kruskal–Wallis test with Dunn's multiple comparisons test, and particle size data using ANOVA with Dunnett's T3 multiple comparisons test. L, large, M, medium, S, small, XS, very small.

Serum cholesterol and the concentrations of LDL‐C, non‐HDL‐C, and HDL‐C were similar in the two groups (Table 1). However, serum TG levels were significantly higher in the low cholesterol absorbers than in the high cholesterol absorbers. Increased TG levels were not associated with statistically significant differences in NMR‐determined total VLDL particle concentration or concentrations of VLDL subclasses between the groups, or VLDL particle size (Fig. 2a). In contrast, despite similar LDL‐C concentrations and even similar concentrations of LDL particles, the high absorbers had less medium‐sized and extra‐small LDL particles than the low cholesterol absorbers, and their LDL particles were larger than in the low cholesterol absorbers (Fig. 2b). The concentration of HDL particles and HDL subclasses did not differ between the high and low cholesterol absorbers, but also HDL particles were larger in the high cholesterol absorbers (Fig. 2c).

**Fig. 3:**
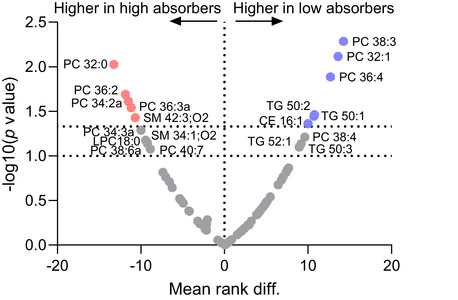
**Differences in low‐density lipoprotein (LDL) lipidome between high and low cholesterol absorbers**. LDL lipid compositions between the low and high cholesterol absorbers were compared using the Mann–Whitney test. The x‐axis shows the mean rank differences between the groups, and the y‐axis the p‐values. The lower dotted line represents p = 0.1 and the upper dotted line represents p = 0.05. Significantly different lipid species are highlighted in blue and red colors in the graph. N = 40 in each group. CE, cholesteryl ester; LPC, lysophosphatidylcholine; PC, phosphatidylcholine; SM, sphingomyelin; TGs, triacylglycerol.

Comparison of the differences in LDL lipidomes in the high and low cholesterol absorbers is shown in Fig. 3. Most of the lipids differing significantly between the groups were LDL surface lipids. Among them was phosphatidylcholine (PC) 36:2, one of the main LDL phospholipids. This lipid was significantly higher in the high cholesterol absorbers. In addition, sphingomyelin (SM) 42:3;O2 (also known as SM 24:2) and PC 32:0 were higher in the high absorbers, whereas PC 32:1, PC 38:3, and PC 36:4 were higher in the low absorbers. The low absorbers also have a higher proportion of TG 50:1 and TG 50:2 species most likely containing 16:0 twice, with one 18:1 or 18:2 (palmitates with oleate or linoleate, respectively) that are associated with increased de novo lipogenesis. The difference in LDL size (Fig. 2b) was not reflected in statistically significant differences in the proportion of phospholipids of the total LDL lipids (low absorbers 13.9% ± 0.56% phospholipids, high absorbers 12.2% ± 0.58% phospholipids, *p* = 0.197).

### Proatherogenic properties of lipoproteins

**Fig. 4:**
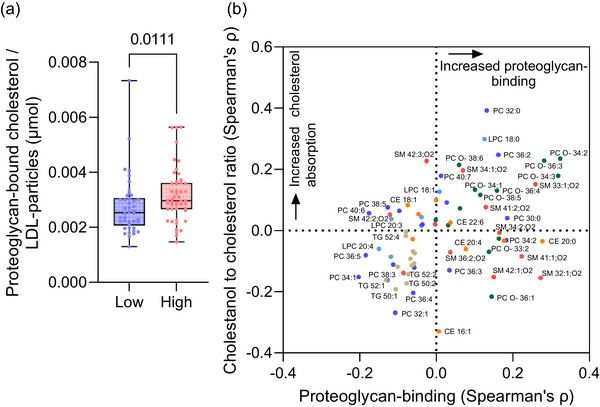
**Proteoglycan‐binding of lipoproteins and its relationship with cholesterol absorption efficiency and low‐density lipoprotein (LDL) lipidome**. (a) The proteoglycan‐binding of serum lipoproteins was determined using a solid‐phase assay, and the bound cholesterol was normalized to the concentration of LDL particles. (b) LDL lipid species and their relationship with proteoglycan binding (x‐axis) and cholestanol to cholesterol ratios (y‐axis). Each dot corresponds to a lipid species colored according to its class. Lipids located in the upper right quadrant are associated with increased proteoglycan‐binding and increased cholesterol absorption. The lipids most significantly associated either directly or inversely to proteoglycan‐binding or cholesterol absorption are shown. CE, cholesteryl ester; LPC, lysophosphatidylcholine; PC, phosphatidylcholine; SM, sphingomyelin; TGs, triacylglycerol.

Variations in the lipidomic profiles of circulating lipoproteins have been linked with differences in their proatherogenic properties [6, 8, 10]. Therefore, we determined whether cholesterol absorption efficiency and the resulting differences in LDL lipidome influence the proteoglycan‐binding capacity of these lipoproteins. Notably, when normalized to the concentration of LDL particles, proteoglycan‐bound cholesterol was higher in high cholesterol absorbers than in low absorbers (Fig. 4a).

To investigate whether the cholesterol absorption efficacy could impact proteoglycan‐binding by influencing the LDL lipidome, we examined the relationship between serum cholestanol/cholesterol ratios, proteoglycan‐binding, and LDL lipidome. We identified that several lipid species, such as PC 32:0, lysophosphatidylcholine 18:0, and other saturated PCs, ether‐linked PCs (PC O–), and nearly all SM species were associated with increased proteoglycan‐binding and elevated cholesterol absorption. In contrast, several PC species with a higher degree of unsaturation, along with the majority of TG species, were associated with reduced proteoglycan‐binding and cholesterol absorption (Fig. 4b).

**Fig. 5:**
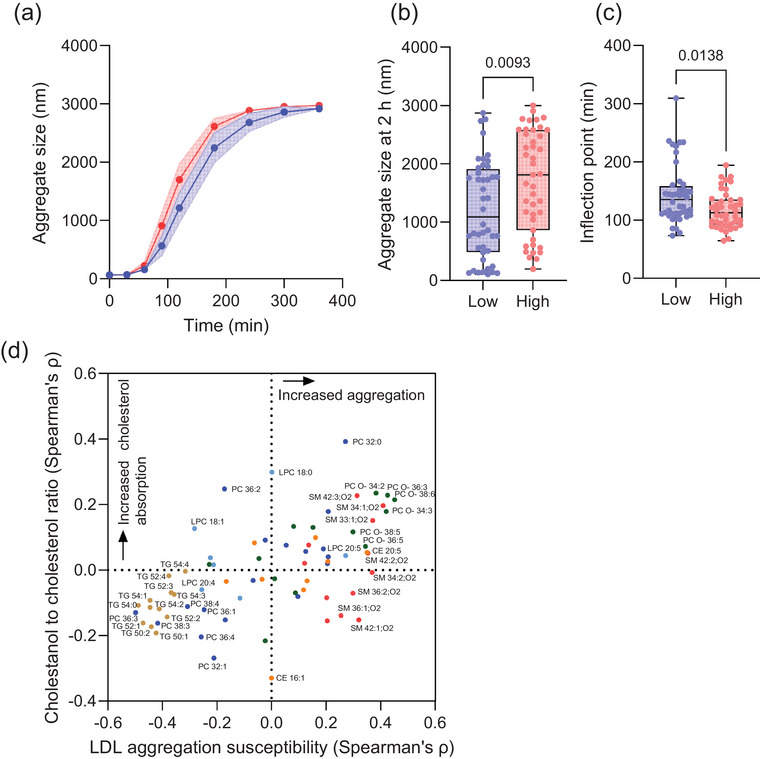
**Low‐density lipoprotein (LDL) aggregation susceptibility and its relationship with cholesterol absorption efficiency and LDL lipidome**. (a) LDL aggregation was induced by human recombinant sphingomyelinase, and aggregation was followed by measuring the aggregate sizes by dynamic light scattering. The time versus aggregate size curves with their 95% confidence intervals are shown for high (red) and low cholesterol absorbers (blue). (b) LDL aggregate sizes at 2 h. (c) The inflection points of the LDL aggregate size versus time curves. The differences between the groups were determined by the Mann–Whitney test. (d) LDL lipid species and their relationship with LDL aggregation susceptibility (aggregate size at 2 h) (x‐axis) and cholestanol to cholesterol ratios (y‐axis). Each dot corresponds to a lipid species colored according to its class. Lipids located in the upper right quadrant are associated with increased aggregation susceptibility and increased cholesterol absorption. The lipids most significantly associated either directly or inversely to LDL aggregation or cholesterol absorption are shown. CE, cholesteryl ester; LPC, lysophosphatidylcholine; PC, phosphatidylcholine; SM, sphingomyelin; TGs, triacylglycerol.

LDL aggregation susceptibility was higher in the high cholesterol absorbers compared to the low absorbers (Fig. 5a). The aggregate size at 2 h was larger, and the inflection point was lower, both indicating a faster rate of LDL aggregation (Fig. 5b,c). Increased LDL aggregation was associated with saturated PCs, PC O‐s, and all SM species (Fig. 5d). In contrast, all TG species were tied to both low cholesterol absorption and reduced LDL aggregation susceptibility. Notably, all lipid species elevated in the high cholesterol absorbers (except for PC 36:2) correlated with increased LDL aggregation susceptibility.

## Discussion

Increased intestinal cholesterol absorption efficiency is associated with higher risk of ASCVD through mechanisms that cannot be attributed to elevated levels of LDL‐C or apoB‐containing lipoproteins in circulation [23, 24]. Our findings demonstrate that high cholesterol absorption efficiency is associated with changes in the lipid composition of LDL particles that drive increased proteoglycan‐binding and susceptibility to aggregation of LDL particles. These two LDL properties are mechanistically linked with lipid accumulation in the arterial wall [5, 36, 37] and predict future cardiovascular events [6, 7]. Based on this, we propose that genetically driven high cholesterol absorption represents a distinct proatherogenic trait that contributes to cardiovascular disease risk by increasing the atherogenic potential of apoB‐lipoproteins.

Cholesterol absorption efficiency did not influence LDL‐C levels, as observed also previously [20, 24, 38], indicating that the mechanisms influencing particle atherogenicity and lipoprotein lipidomes operate independently of circulating LDL‐C levels. Liver‐mediated homeostatic regulation of cholesterol appears to be able to maintain stable cholesterol levels in circulation despite alterations in cholesterol absorption efficiency [23]. Increased influx of cholesterol suppresses sterol regulatory element binding proteins and lowers hepatic cholesterol synthesis [39], whereas low cholesterol absorption is associated with increased reverse cholesterol transport from peripheral tissues to liver [40] but also bile excretion [41].

Despite similar cholesterol concentrations, low cholesterol absorbers exhibited smaller LDL and HDL particles, increased circulating TGs, dietary saturated fatty acids, and higher BMI. In accordance, overweight [42] and increased TG levels [43, 44] have previously been linked with low cholesterol absorption. This profile is distinctive of metabolic dyslipidemia characterized by small LDL and HDL particles resulting from the combined action of cholesteryl ester transfer protein (CETP) and hepatic lipase. CETP facilitates the exchange of TGs from VLDL to LDL and HDL, effectively enriching these particles with TGs that are subsequently hydrolyzed by hepatic lipase, reducing the size of the LDL and HDL [45]. This process is enhanced in overweight individuals, and when hepatic TGs are increased, it aligns with the characteristics of our low absorption group [43, 44].

Low cholesterol absorbers have been shown to have increased bile acid and cholesterol synthesis [41]. Notably, a reduction in bile acids was recently shown to suppress overeating in mice [46], suggesting a potential link between bile acid metabolism and appetite regulation in which enterocytes could play a part through the lipid‐sensing gut–brain axis [47]. These observations combined with our congruent findings highlight the possibility that individuals that have low cholesterol absorption, increased cholesterol synthesis, and bile acid secretion experience increased appetite for saturated fatty acids, explaining the frequently observed increase in circulating TGs upon reduced cholesterol absorption [43, 44].

Although circulating cholesterol levels are stabilized despite differences in cholesterol absorption, we observed qualitative differences in the lipidomes of LDL particles between high and low cholesterol absorbers. Differences were observed mainly in phospholipids reminiscent of newly synthetized lipid species such as LDL‐PC 32:0 (16:0/16:0) in high cholesterol absorbers (Fig. 3). Increased cholesterol absorption into the intestinal enterocytes leads to enrichment of both unesterified cholesterol and CE within chylomicrons [48], which could promote phospholipid and sphingolipid synthesis in enterocytes. These freshly synthetized phospholipid and sphingolipid species can subsequently be transferred from chylomicrons to LDL and HDL in circulation [49], which could explain their increase in LDL particles among high cholesterol absorbers.

Previous studies have demonstrated that conformational changes in apoB‐100 driven by the lipid composition and size of the particles determine proteoglycan‐binding, with small dense LDL particles exhibiting the highest affinity for proteoglycans on a per‐particle basis [50, 51, 52]. However, our current data indicate that high cholesterol absorbers, despite having fewer small LDL particles, show increased bound cholesterol per LDL particle. This observation aligns with reports that ezetimibe, which reduces cholesterol absorption, reduces the proteoglycan‐binding of apoB‐containing lipoproteins [53]. It is important to note that our solid‐phase proteoglycan‐binding assay quantifies the total amount of *cholesterol* bound to proteoglycans. Consequently, although smaller LDL particles may have higher affinity per particle for proteoglycans, the larger LDL particles containing more cholesterol molecules per particle can ultimately lead to increased accumulation of cholesterol [54].

LDL from the high cholesterol absorbers was more aggregation‐prone than LDL from low cholesterol absorbers independently of circulating cholesterol levels. Several surface phospholipids of LDL particles were found to differ between the high and the low cholesterol absorbers and associate with differences in LDL aggregation susceptibility and proteoglycan binding. Previous studies have shown that LDL particles enriched with SM tend to be more prone to aggregation than those enriched with PC due to alterations in the conformation of apoB‐100 on LDL surface [6, 55]. Interestingly, the inhibition of the conformational changes in apoB‐100 reduces aggregation and decreases atherosclerosis [13, 56]. In the present study all SM species were associated with increased LDL aggregation susceptibility.

When considering the effect of PC lipids in the atherogenicity of the LDL particles, an interesting pair is PC 32:0 and PC 32:1. PC 32:0, most likely containing two 16:0 fatty acids, was higher in the high absorbers and associated with increased LDL aggregation susceptibility and proteoglycan‐binding. In contrast, PC 32:1, most likely containing one 16:0 and one 16:1 (palmitoleate) fatty acid, was elevated in the low absorbers and correlated with reduced LDL aggregation susceptibility and proteoglycan binding.

PC 32:0 is one of the lipids preferentially accumulating in atherosclerotic lesions [57], where its proportion in extracellular lipoproteins is more than 5‐fold higher than in circulating lipoproteins [58]. The PC 32:0 harbors fatty acid 16:0, which has been shown to cause endoplasmic reticulum stress, lipotoxic cell death, and inflammation [59, 60], whereas the 16:1, particularly when carried in CEs, has been associated with increased hepatic lipogenesis [59] and improved insulin sensitivity [61, 62].

**Fig. 6:**
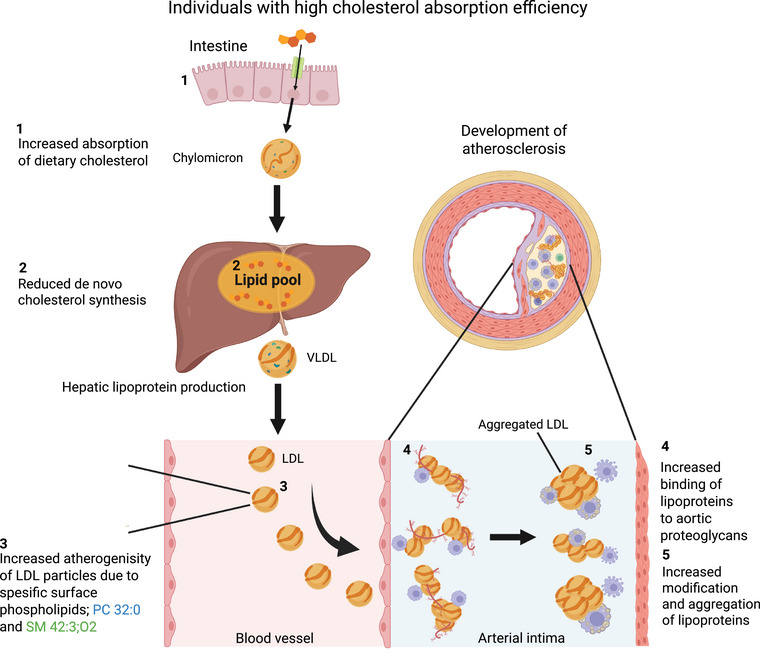
**A summary figure offers a potential explanation to how increased cholesterol absorption increases the risk of atherosclerotic cardiovascular disease (ASCVD) compared to low cholesterol absorbers**. In the figure: (1) individuals with higher cholesterol absorption experience increased uptake of dietary cholesterol from the small intestine. (2) To counteract this rise in circulating cholesterol, de novo cholesterol synthesis in the liver is reduced, thereby maintaining similar cholesterol levels to those in low cholesterol absorbers. (3) The low‐density lipoprotein (LDL) particles in the circulation exhibit altered lipid composition, being enriched in phosphatidylcholine (PC) 32:0 and sphingomyelin (SM) 42:3;O2. (4) Upon migrating to the arterial intima, these LDL particles, due to their altered lipid composition, are more prone to binding to matrix proteoglycans, resulting in their retention within the intima. (5) Furthermore, the altered lipid composition promotes aggregation of LDL particles in the intima. These processes are accentuated in individuals with high cholesterol absorption, thereby contributing to an increased risk of ASCVD, as previously reported. VLDL, very low‐density lipoprotein.

Our study provides information on the influence of cholesterol absorption efficiency on the lipid composition, aggregation tendency, and proteoglycan‐binding affinity of LDL particles (Fig. 6). By integrating lipidomic analyses with functional assays, we have captured key mechanistic aspects of the atherogenicity of LDL particles. However, some limitations should be acknowledged. Although our cohort size allows meaningful comparisons, larger studies in multiethnic populations would enhance generalizability. We controlled major confounders, such as diet and age, but other factors, such as individual inflammatory status, menopause or potential hormone replacement therapy, or genetic variability, could still influence LDL properties. The methodological approaches used to assess LDL aggregation and proteoglycan binding, though well‐established, may not fully replicate in vivo conditions. In addition, our lipidomic analysis does not include all minor lipids carried in LDL, including ceramides, which have been suggested to be markers of ASCVD risk [63, 64]. Despite these considerations, our findings contribute to a growing body of evidence on the interplay between cholesterol metabolism and lipoprotein functions. The present study indicates that high cholesterol absorption represents a distinct proatherogenic trait that operates independently of plasma LDL‐C concentrations by increasing the atherogenicity of the LDL particles. The findings underscore the importance of assessing proatherogenic factors—such as proteoglycan‐binding, LDL aggregation susceptibility, and cholesterol absorption—in addition to LDL‐C levels to more comprehensively evaluate ASCVD risk.

## Author contributions

**Katariina Öörni**: Writing—original draft; writing—review and editing; conceptualization; data curation; formal analysis; funding acquisition; project administration; supervision; visualization. **Lauri Äikäs**: Writing—review and editing; investigation; methodology; visualization. **Maija Ruuth**: Writing—review and editing; formal analysis; investigation; methodology. **Feven Tigistu‐Sahle**: Writing—review and editing; formal analysis; investigation; methodology. **Reijo Käkelä**: Writing—review and editing; formal analysis; resources; supervision. **Ingmar Wester**: Writing—review and editing; conceptualization; funding acquisition; resources. **Helena Gylling**: Writing—original draft; writing—review and editing; conceptualization; data curation; formal analysis; funding acquisition; investigation, project administration; resources; supervision. **Piia Simonen**: Writing—original draft; writing—review and editing; conceptualization; data curation; formal analysis; funding acquisition; project administration; resources; supervision.

## Conflict of interest statement

Katariina Öörni and Maija Ruuth are inventors in a patent “Measurement of LDL instability” Patent number: US 11442072. The other authors report no conflicts.

## Funding information

This work was supported by Jenny and Antti Wihuri Foundation. Academy of Finland (nos. 315568 and 332564), Finnish Foundation for Cardiovascular Research, Sigrid Jusélius Foundation, Novo Nordisk Foundation (no. NNF19OC0057411), and Raisio Nutrition Ltd.

## Data Availability

The data that support the findings of this study are available on request from the corresponding author. The data are not publicly available due to privacy or ethical restrictions.
